# Successful reinterventional drainage via endoscopic ultrasound-guided cannulation with anastomotic identification

**DOI:** 10.1055/a-2505-9500

**Published:** 2025-02-11

**Authors:** Daisuke Namima, Toshio Fujisawa, Yusuke Takasaki, Ko Tomishima, Shigeto Ishii, Hideki Kobara, Hiroyuki Isayama

**Affiliations:** 1 Department of Gastroenterology, Graduate School of Medicine, Juntendo University, Tokyo, Japan; 2 Department of Gastroenterology and Neurology, Kagawa University, Kagawa, Japan

The rate of reinterventions, including stent or stone management via anastomosis, has increased following endoscopic ultrasound (EUS)-guided biliary drainage/anastomosis 1 2 3 4 5 . Stent dislodgement presents significant challenges due, in particular, to difficulty locating the anastomotic site and recannulating the anastomotic tract after EUS-guided hepaticogastrostomy (EUS-HGS). Recannulation using a duodenoscope, which differs from the original EUS-HGS equipment, can complicate the procedure owing to challenges in identification of the anastomotic site and adjustment of the insertion angle. Therefore, we used the original EUS scope for reinterventions following EUS-HGS.

A 32-year-old man underwent EUS-HGS for biliary obstruction following a duodenectomy for a duodenal ulcer. Subsequently, the patient presented with obstructive jaundice due to the dislodgement of a plastic stent ( Fig. 1 ). Using an EUS scope (UCT-740; Fujifilm Corp., Tokyo, Japan), the anastomotic site was identified based on mucosal traction observed on the lesser gastric curvature ( Fig. 2 ). Despite multiple attempts to recannulate the bile duct from this site using a catheter, EUS was used to visualize the anastomotic tract as a hypoechoic band within the liver parenchyma ( Fig. 3 ). Under EUS guidance, the catheter was advanced into the anastomotic tract, and a 0.025-inch guidewire was successfully inserted into the bile duct. Subsequently, a new plastic stent was placed into the bile duct through the anastomosis ( Video 1 ).

**Fig. 1:**
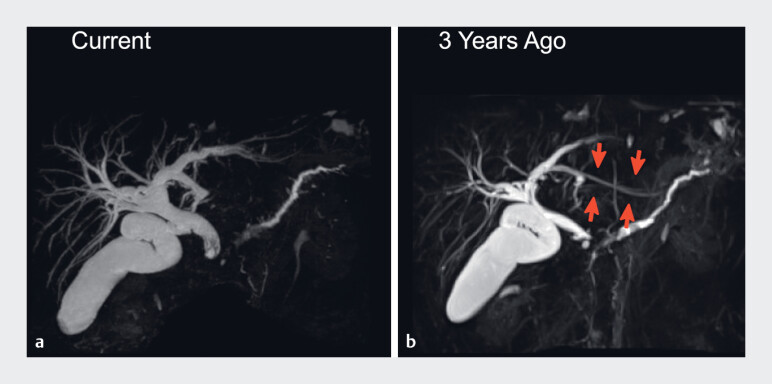
Comparison of current and previous magnetic resonance cholangiopancreatography (MRCP) findings. **a** The current MRCP. **b** The previous MRCP. The current MRCP demonstrated bile duct dilation compared with findings from 3 years previously. The plastic stent placed 3 years previously (red arrows) was no longer visible.

**Fig. 2:**
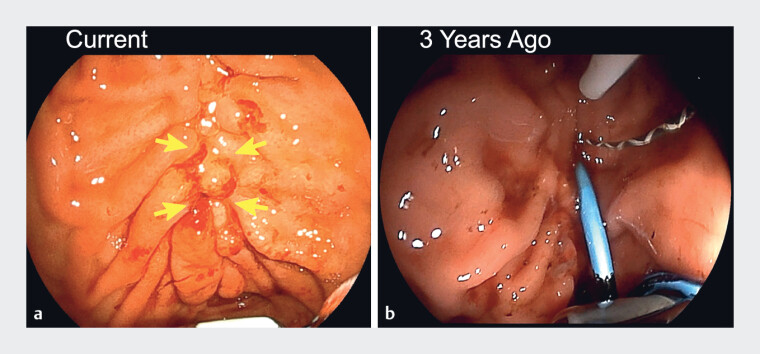
Comparison of endoscopic findings. **a** The current endoscopic image showed mucosal traction along the lesser curvature of the gastric body, indicated by the yellow arrow. **b** The same region 3 years earlier showed a plastic stent.

**Fig. 3:**
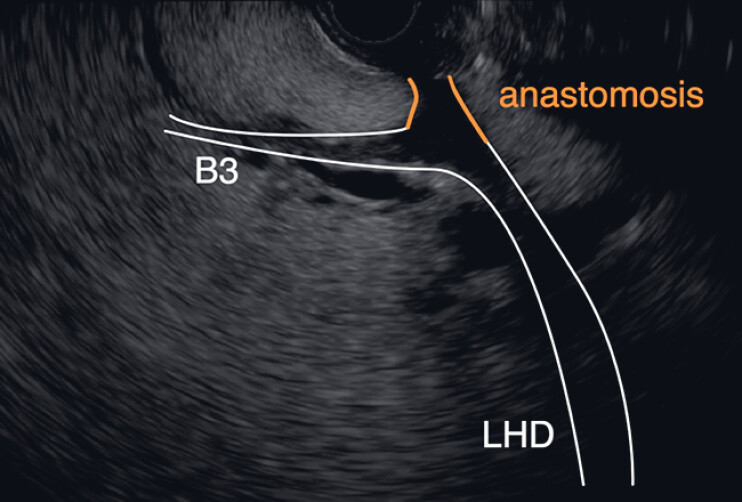
Endoscopic ultrasound findings. A hypoechoic area consistent with the anastomosis was identified in continuity with the bile duct. LHD, left hepatic duct.

A plastic stent was successfully placed in the bile duct through endoscopic ultrasound-guided cannulation.Video 1

This case demonstrates that EUS under endoscopic visualization is a helpful means of identifying the anastomotic tract. EUS-guided recannulation into the anastomotic tract is effective when the anastomotic site is lost due to stent or guidewire dislodgement. The use of the EUS scope proved beneficial for reintervention following EUS-HGS, enabling directional adjustment and EUS-guided recannulation through the anastomosis.

Endoscopy_UCTN_Code_TTT_1AS_2AH

## Notes

Endoscopy E-Videos https://eref.thieme.de/e-videos
*E-Videos* is an open access online section of the journal *Endoscopy* , reporting on interesting cases and new techniques in gastroenterological endoscopy. All papers include a high-quality video and are published with a Creative Commons CC-BY license. Endoscopy E-Videos qualify for HINARI discounts and waivers and eligibility is automatically checked during the submission process. We grant 100% waivers to articles whose corresponding authors are based in Group A countries and 50% waivers to those who are based in Group B countries as classified by Research4Life (see: https://www.research4life.org/access/eligibility/ ). This section has its own submission website at https://mc.manuscriptcentral.com/e-videos .
